# Heavy Metal Removal from Wastewater Using Poly(Gamma-Glutamic Acid)-Based Hydrogel

**DOI:** 10.3390/gels10040259

**Published:** 2024-04-11

**Authors:** Fujie Chen, Yanbin Zhao, Hang Zhao, Xuan Zhou, Xiuying Liu

**Affiliations:** 1 School of Chemistry and Chemical Engineering, Wuhan Textile University, Wuhan 430200, China; whcfj@wtu.edu.cn (F.C.);; 2 Hubei Key Laboratory of Biomass Fibers and Eco-Dyeing & Finishing, Wuhan Textile University, Wuhan 430200, China; 3 School of Chemistry and Environmental Engineering, Wuhan Institute of Technology, Wuhan 430205, China

**Keywords:** heavy metal, adsorption, hydrogel, water treatment, polymer, poly(gamma-glutamic acid)

## Abstract

The removal of toxic heavy metal ions from wastewater is of great significance in the protection of the environment and human health. Poly(gamma-glutamic acid) (PGA) is a non-toxic, biodegradable, and highly water-soluble polymer possessing carboxyl and imino functional groups. Herein, water-insoluble PGA-based hydrogels were prepared, characterized, and investigated as heavy metal adsorbents. The prepared hydrogels were recyclable and exhibited good adsorption effects on heavy metal ions including Cu^2+^, Cr^6+^, and Zn^2+^. The effects of adsorption parameters including temperature, solution pH, initial concentration of metal ions, and contact time on the adsorption capacity of the hydrogel for Cu^2+^ were investigated. The adsorption was a spontaneous and exothermic process. The process followed the pseudo-first-order kinetic model and Langmuir isotherm model, implying a physical and monolayer adsorption. The adsorption mechanisms investigation exhibited that Cu^2+^ adsorbed on the hydrogel via electrostatic interactions with anionic carboxylate groups of PGA in addition to the coordination interactions with the –NH groups. Importantly, the PGA hydrogel exhibited good reusability and the adsorption capability for Cu^2+^ remained high after five consecutive cycles. The properties of PGA hydrogel make it a potential candidate material for heavy metal ion removal in wastewater treatment.

## 1. Introduction

Most heavy metal ions, even at low concentrations, are toxic and can have detrimental effects on the environment and human health [1]. Industries such as non-ferrous metallurgical industry, fine chemical, paper, engineering, paint, dye, petrochemical, textile, and pharmaceutical, inevitably lead to wastewater with excess concentration of heavy metals [2,3,4]. Removal of heavy metals from wastewater is of great significance [5]. A variety of technologies including adsorption, extraction, chemical precipitation, ion exchange, electrodialysis, reverse osmosis, and redox have been developed for the removal [5,6,7,8,9,10,11]. For example, Yudaev and Chistyakov systematized the works on the extraction process, the classes of chelating extractants for metals, and the efficiency and selectivity of the extractants in the recovery of various metals from industrial wastewater, soil, spent raw materials, and the separation of metal mixtures [11]. The technology of adsorption is based on the physical or chemical interaction between the adsorbent and the heavy metal ions [5,12,13,14]. It is widely studied due to the relatively simple operation process, the flexibility in the design of adsorbents, and the high removal efficiencies [5].

The adsorbents are various, and Wang et al. categorized them into biosorbents (e.g., agriculture waste biochar/activated carbon, algae, and bacteria) and abiotic adsorbents (e.g., polymers, microtubes, metal-organic frameworks, minerals, clays, and coal) [6,7,15]. Hydrogels are soft matter with a three-dimensional structure created via the cross-linking of synthetic or natural polymers. They have superior applications for heavy metal removal, due to their excellent properties, such as high functionality, high porosity, handling, ease of preparation, and easy recovery [16]. Many documents have summarized recent progress in the use of hydrogel adsorbents for the removal of heavy metal ions and focused on the adsorption performances of the hydrogels [16,17,18,19,20]. Developments in the synthesis of hydrogel-based adsorbent materials have also been summarized. Hydrogels are generally synthesized via chemical or physical cross-linking [16,19]. The chemical route of cross-linking can be via free radical polymerization, high-energy irradiation, grafting reactions, and reaction of functional groups. The physical route of cross-linking can be via freeze-thaw, self-assembling, instantaneous gelation, ionotropic gelation, or inverse emulsion method.

Hydrogels function as excellent adsorbents in heavy metal removal processes by binding metal ions with various functional groups in their polymeric networks. The adsorptions rely on physical and chemical interactions, such as electrostatic interaction, coordination interaction, hydrophobic interaction, and ion exchange, depending on the surface functional moieties of hydrogels. According to previous documents, electrostatic interactions are the dominant adsorption force for heavy metal abstraction in various hydrogels [19]. The paramount functional groups in hydrogels for ion adsorption are generally classified into three groups: (a) nitrogen-containing functional groups, such as the amine group (–NH_2_), amide group (–CONH), and quaternary ammonium group [–N^+^(CH_3_)_3_]; (b) oxygen-containing functional groups, such as the hydroxyl (–OH) and carboxyl group (–COOH); and (c) sulfur-containing functional groups, such as the thiol (–SH) and sulfonic acid group (–SO_3_H). Other functional groups include the amidoxime group (–C(NH_2_)=N–OH), phosphate-containing functional groups (phosphine, phosphate, and phosphoramide), and chelating groups such as the nitrogen- and oxygen-containing functional groups present in the aminopolycarboxylic acid structure [19].

Hydrogel-forming polymers include natural polymers such as cellulose [21], chitosan and alginate [17,21,22,23,24,25], and synthetic polymers such as polyvinyl alcohol (PVA) [26], polypyrrole [20], aminated polyacrylonitrile [27], polypyrrole-polyaniline [20], and poly(acrylic acid-co-acrylamide) [16]. Chitosan and alginate-based hydrogels are widely studied for the adsorption of metal ions. Composite hydrogels are also well documented. Alginate and chitosan can be incorporated with other components to prepare hydrogels or nanocomposite materials with different efficiencies to remove metal ions and dyes [17].

γ-polyglutamic acid (PGA) is a biodegradable, non-toxic, and highly water-soluble polymer derived from glutamic acid and possesses carboxyl functional groups (–COOH) along the polymer side chains and imino groups (–NH) along the backbones. These functional groups have a high binding affinity for heavy metal ions [14]. However, PGA in aqueous form as an adsorbent faces difficulty in separating the heavy metal-accumulating PGA from water [12,13,28,29,30]. PGA in the form of nanoparticles can be separated from water; however, to achieve the separation of nanoparticles, ultrafiltration technology using nanomembranes is required [31]. Wang and co-workers reported a hybrid alginate-polyglutamic acid hydrogel for the removal and recovery of rare earths (III) from dilute solution. Doping PGA into calcium alginate can significantly enhance the adsorption capacity and the selectivity of rare earths from non-rare earths. The maximum adsorption capacity obtained for Nd(III) was 1.65 mmol/g [32]. Yin et al. designed a combination of γ-polyglutamic acid, polylysine (ε-PL), and tannin and prepared a composite PGA-PL-tannin gel. The removal rate of Cr(VI) by this gel exceeded 90% [33].

**Figure 1:**
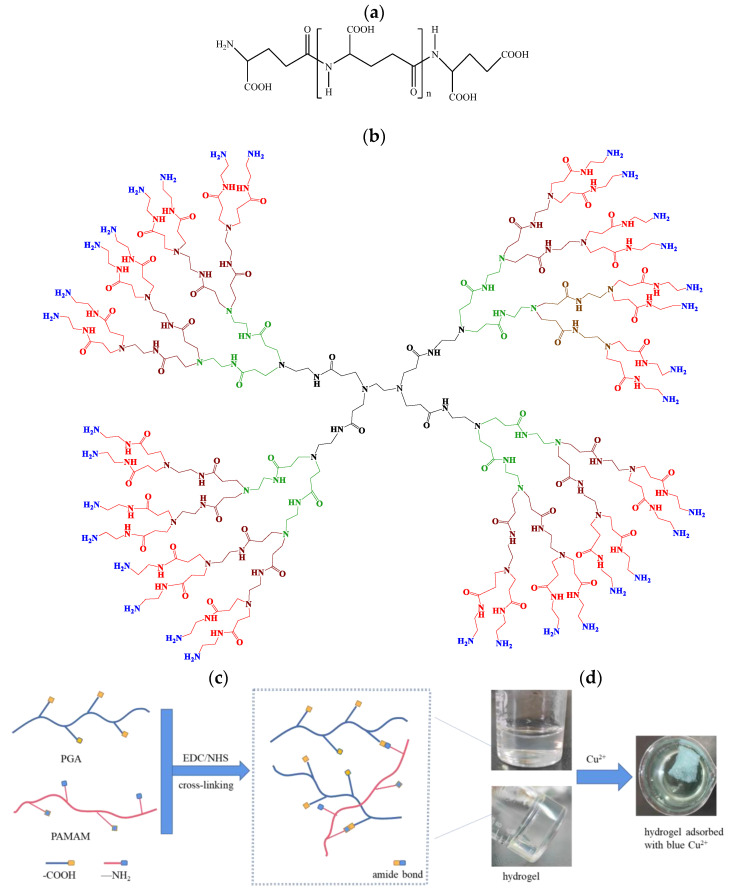
(**a**) Structure of γ-PGA; (**b**) structure of third-generation PAMAM [34]; (**c**) preparation scheme of water-insoluble hydrogel by cross-linking between γ-PGA and PAMAM; (**d**) images of hydrogel before and after adsorption of Cu^2+^.

In this study, we aim to prepare a PGA-based hydrogel that is water-insoluble and recyclable, compared to an aqueous PGA adsorbent. It avoids the problem of difficulty in separating heavy metal-accumulating PGA from water. Its performance in heavy metal removal is investigated. A third-generation polyamindoamine (PAMAM) dendrimer with several terminal amino functional groups that are reactive toward PGA is used as the cross-linking reagent, as shown in Figure 1. Cu^2+^ was used as the heavy metal model pollutant. The hydrogel is characterized and the effect of adsorption parameters on adsorption is investigated. The adsorption isotherms, adsorption kinetics, thermodynamics models, and the possible adsorption mechanism for Cu^2+^ ions onto PGA hydrogel are analyzed. The reusability of PGA hydrogel is also explored. 

## 2. Results and Discussion

### 2.1. Characterizations of the Hydrogel

**Figure 2:**
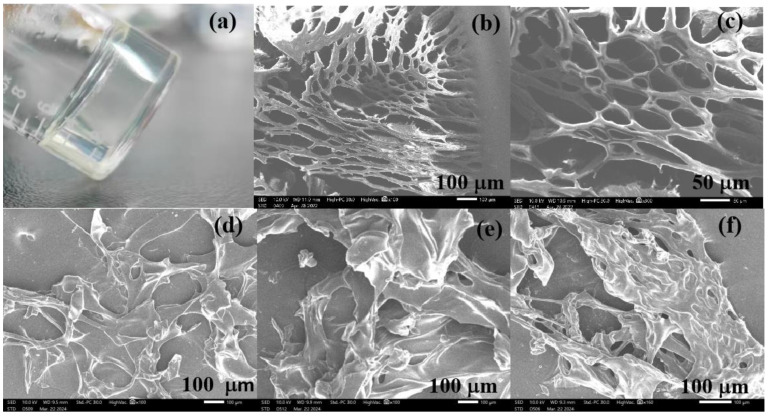
(**a**) Real image of the hydrogels; (**b**,**c**) SEM images of the hydrogels before metal ion adsorption under different magnifications; (**d**–**f**) SEM images of the hydrogels after adsorption of Cu^2+^, Zn^2+^, and Cr^6+^ ions, respectively.

**Figure 3:**
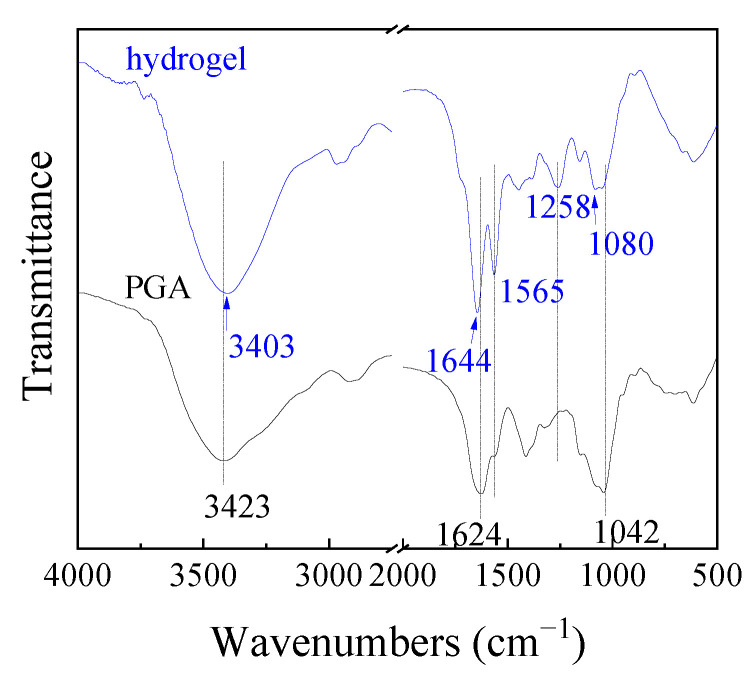
FTIR of PGA and the hydrogel.

**Figure 4:**
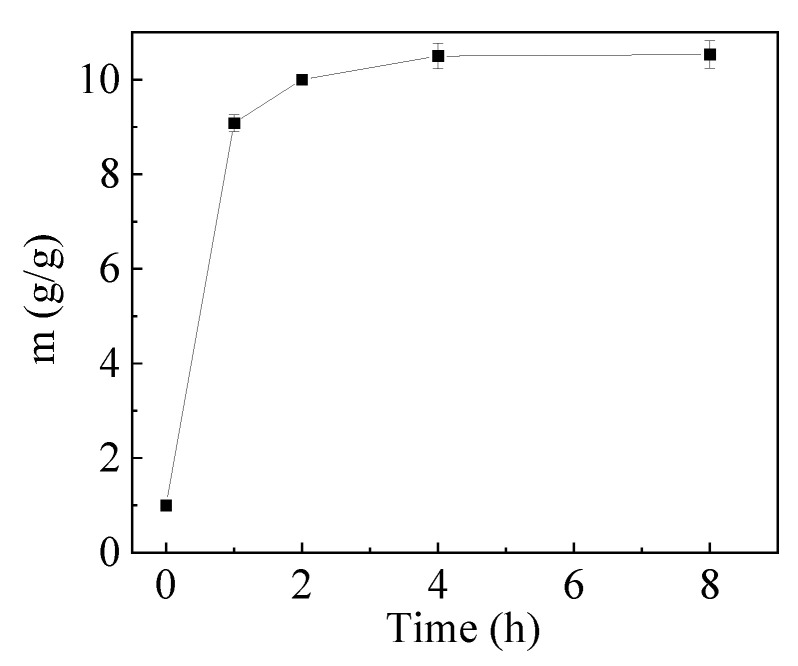
Swelling ratio of the hydrogel.

The hydrogel was prepared, and the real image is shown in Figure 2a. The SEM images in Figure 2b,c exhibited that the hydrogel had a porous and regular network structure. The porous structure makes it possible for the metal ions to enter the hydrogel. The network structure indicated that cross-linking was formed between PGA and PAMAM and led to the water-insolubility of the hydrogel. SEM of the hydrogels after adsorption of Cu^2+^, Zn^2+^, and Cr^6+^ ions are shown in Figure 2d–f, which revealed that the hydrogel surfaces were irregular and had lower porosity than the hydrogel before metal ion adsorption. This is because the electrostatic repulsions decline in the network of hydrogels after the anionic groups of the hydrogels absorb the cationic metal ions, as reported by Yang et al. [35]. Another reason is due to metal ions occupying the pores [36]. To confirm the chemical cross-linking, FTIR of the hydrogel and PGA were compared, as shown in Figure 3. In the infrared spectrum of PGA, the peak at 3423 cm^−1^ belongs to the stretching vibrations of the carboxylic O–H and imino N–H, and the absorption band at 1624 cm^−1^ can be assigned to N–H shearing vibration. In contrast to PGA, the corresponding peaks of the hydrogel appear at 3403 cm^−1^ and 1644 cm^−1^, respectively. The peak shifts indicated that part of the –COOH and –NH groups participated in the cross-linking between PGA and PAMAM. The hydrogel exhibits a new strong peak at 1565 cm^−1^, which corresponds to the solid N–H peak or N–H shear vibration of secondary amide. In addition, two other new peaks are observed at 1258 and 1080 cm^−1^, attributable to the stretching vibrations of the C–O and C–N bonds of the secondary amide. These results imply that the amide groups (–NHCO–) formed upon cross-linking. The BET test results showed that the specific surface area of the hydrogel is very small, implying that the specific surface area had little effect on the adsorption of copper ions. The hydrogel was immersed in water for 8 h and it was insoluble in water. As shown in Figure 4, the swelling ratio of the hydrogel reached 10.53 g/g, indicating the excellent hydrophilicity of the PGA hydrogel. The hydrogels with good swelling properties provided diffusion channels for heavy metal ions, which facilitated the binding of metal ions to the adsorption sites inside the hydrogel materials [37]. Chowdhury et al. reported that the degree of swelling of PVA hydrogels is about 370% and the removal of copper by PVA hydrogels is about 7 mg/g [38]. According to Romal et al., the swelling capacities of chitosan hydrogel and Mannich base-modified chitosan (CS-MB) hydrogel in unbuffered distilled water after 4 min reached 25.7 g/g and 8.09 g/g, respectively. CS-MB hydrogel showed a Cu^2+^ adsorption capacity of 12.0 mg/g. However, no data were provided by them about the adsorption capacity of chitosan hydrogel [39]. However, Dai and co-workers reported that the hydration rate and the copper(II) adsorption capacity of chitosan hydrogel reached 94.07% and 60 mg/g, respectively [40]. What is more, the water content of the swelling carboxymethylated chitosan (CMC) hydrogel beads is 95.5% and its equilibrium Cu(II) uptake is as high as about 130 mg/g [41]. Cellulose hydrogel also has a great swelling ability of 4650% and a high adsorption capacity of 28.4 mg/g for copper ions [37]. In contrast, the swelling ability of the PGA hydrogel is higher than that of PVA, and lower than that of chitosan, CS-MB, cellulose, and CMC. 

### 2.2. Adsorption Performance of PGA Hydrogels

#### 2.2.1. Adsorption Kinetics

**Figure 5:**
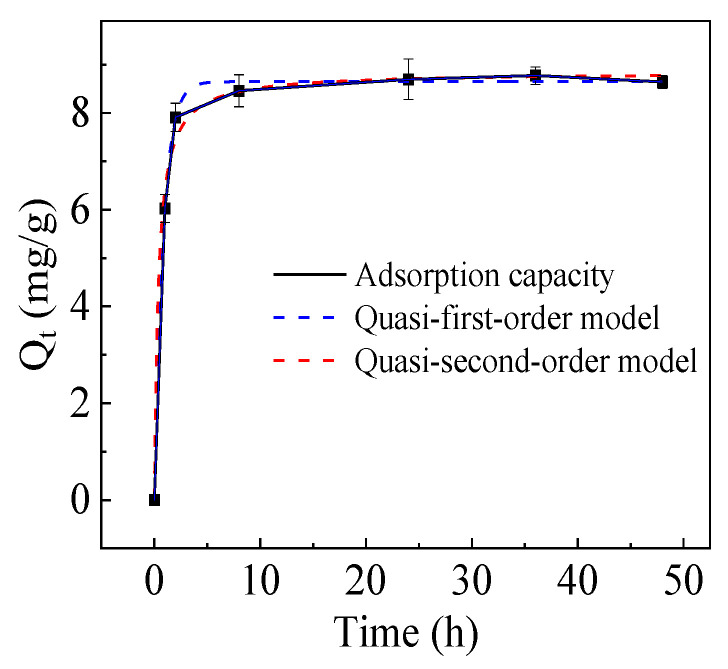
Adsorption kinetic of Cu^2+^ on the hydrogel.

The adsorption capacity of the hydrogel for Cu^2+^ with time is shown in Figure 5. It increased rapidly within 2 h, then slowly, and reached its equilibrium value of 8.6 mg/g within 24 h. The value is equivalent to that of polyvinyl alcohol [38] and the composite adsorbent of collodion membrane cross-linked poly-γ-glutamic acid [42], and lower than that of PGA in aqueous form as an adsorbent. It is also lower than cellulose and chitosan, which have high swelling capacities. To investigate the mechanism of the adsorption process, the adsorption kinetic data were analyzed using pseudo-first-order and pseudo-second-order models, and the corresponding equations are as follows:

(1) $$Q_{t}=q_{e1}\times (1-e^{-k_{1}t})$$

(2) $$Q_{t}=\frac{q_{e2}^{2}k_{2}t}{1+q_{e2}k_{2}t}$$

where *Q_t_* (mg/g) represents the adsorption capacity of the hydrogel at time *t*. *q_e_*_1_ and *q_e_*_2_ (mg g^−1^) are the equilibrium adsorption capacity of the hydrogels calculated by pseudo-first-order and pseudo-second-order kinetic models, respectively. *k*_1_ (min^−1^) and *k*_2_ (g mg^−1^ min^−1^) represent the adsorption rate constants of the pseudo-first-order model and pseudo-second-order model, respectively.

The fitted curves of the adsorption kinetic equation and the fitted parameters are demonstrated in Figure 5 and Table 1, respectively. Based on the correlation coefficients (R^2^) of the equations, the adsorption process followed the pseudo-first-order (R^2^ = 0.9989) kinetic better than the pseudo-second-order kinetic (R^2^ = 0.9261), illustrating that physical adsorption is dominant in the adsorption process [43]. In addition, the theoretical equilibrium adsorption capacity calculated by the quasi-first-order model (*q_e_*_1_) was closer to the experimental equilibrium adsorption capacity (*Q_e_*) than that of the quasi-second-order model (*q_e_*_2_), confirming that the adsorption of Cu^2+^ onto the hydrogel was controlled by the physical adsorption [43].

#### 2.2.2. Adsorption Isotherm

**Figure 6:**
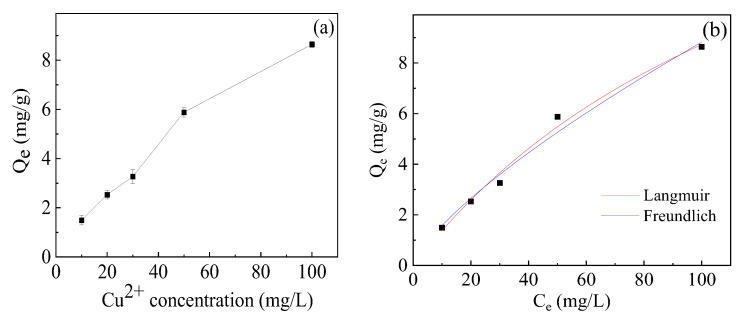
(**a**) Adsorption isotherm of Cu^2+^ and (**b**) fitted curves of Langmuir and Freundlich adsorption isotherm models.

To further study the adsorption mechanism, the adsorption isotherm for Cu^2+^ adsorption on hydrogel was investigated. As shown in Figure 6a, *Q_e_* increased with the increase in initial Cu^2+^ concentration. The Langmuir and Freundlich isotherms that are the most commonly used are applied to interpret the adsorption behavior. Equations (3) and (4) are the formulae for the Langmuir and Freundlich models.

(3) $$Q_{e}=\frac{K_{L}Q_{m}C_{e}}{1+K_{L}C_{e}}$$

(4) $$Q_{e}=K_{F}C_{e}^{\frac{1}{n}}$$

where *Q_m_* (mg g^−1^) is the theoretical maximum adsorption capacity; *K_L_* (L mg^−1^) and *K_F_* (L^n^ mg^n−1/n^ g^−1^) are Langmuir and Freundlich constants, respectively; 1/n is the surface heterogeneity of the adsorbent. The fitted curves and the parameters obtained from two isotherm models are shown in Figure 6b and Table 2, respectively. The adsorption process is better suited to the Langmuir model with higher R^2^ values (R^2^ = 0.9863) than the Freundlich isotherm (R^2^ = 0.9781), implying that the adsorption of copper ions by hydrogel is mainly through single-layer adsorption [44].

#### 2.2.3. Adsorption Thermodynamics

The thermodynamics study is helpful to understand the adsorption process better; the thermodynamic parameters from the experiment at different temperatures are shown in Table 3. The negative value of Δ*H*^0^ of adsorption Cu^2+^ represents that the adsorption process is exothermic. The negative values of Δ*G*^0^ at all tested temperatures represent the spontaneous nature of adsorption. In addition, the increase in Δ*G*^0^ with the increase in temperature exhibits that the adsorption becomes more favorable at the lower temperature.

#### 2.2.4. Effect of Solution pH on Adsorption

**Figure 7:**
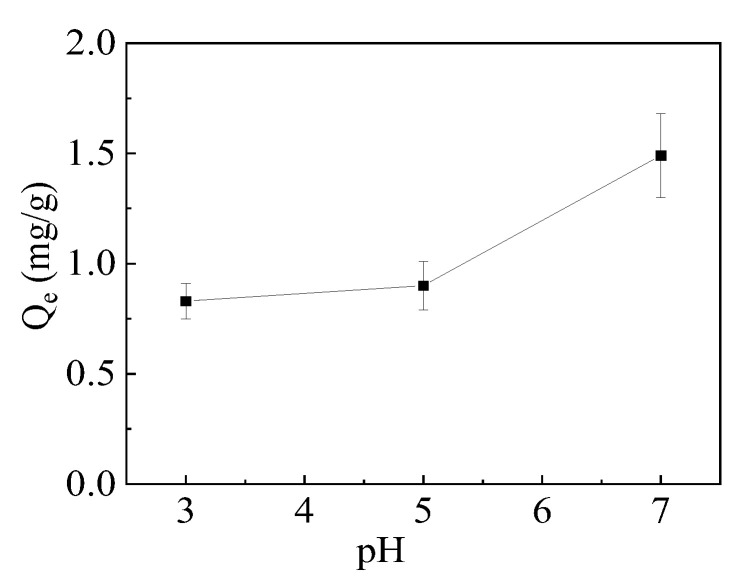
Effect of solution pH on adsorption.

As shown in Figure 7, the adsorption capacity of hydrogel for Cu^2+^ increases with the increase in solution pH from 3 to 7. While the solution pH continues to increase and the value is over 7, the precipitation of Cu(OH)_2_ occurs. At higher solution pH (pH > pK_a_~4), –COOH groups in PGA polymer side chains will ionize to form the anionic carboxylate (–COO^−^) groups because the pK_a_ of PGA is 4.09 [45]. The negatively charged –COO^−^ groups are significantly stronger ligands than –COOH for metal ion binding [46]. Therefore, the increased adsorption capacity was mainly attributed to the electrostatic interaction between –COO^−^ and the cationic ion Cu^2+^. In addition, at higher solution pH, the imino –NH groups in the polymer backbone are deprotonated. The deprotonated –NH is an electron-donating group that had higher adsorption affinity towards the cationic ion Cu^2+^ and adsorbed Cu^2+^ via the coordination interaction, compared to the protonated –NH_2_^+^ group [1].

#### 2.2.5. Effect of the Mass of the Adsorbent on Adsorption

**Figure 8:**
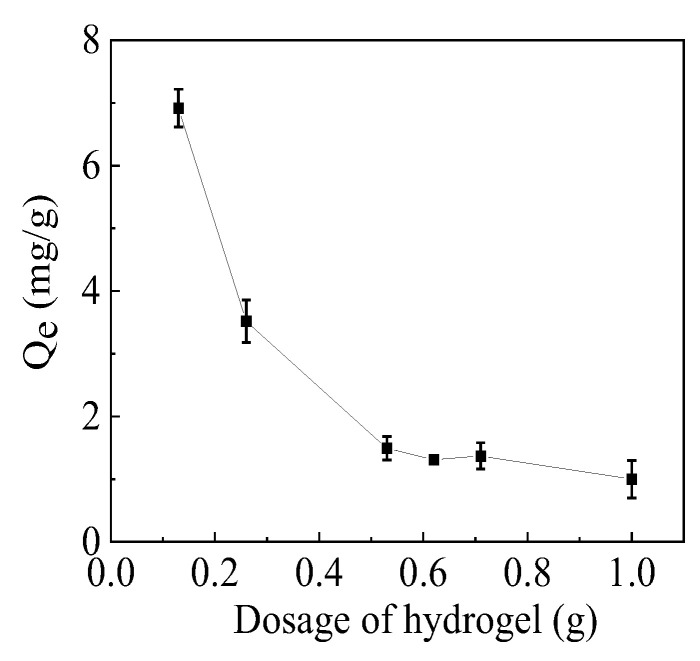
Effect of the adsorbent dosage on adsorption.

The effect of the mass of the adsorbent on the adsorption capacity is shown in Figure 8. The adsorption capacity decreased with the increase in adsorbent weight, consistent with β-cyclodextrin-based adsorbent and poly(methacrylic acid)/zeolite hydrogel composites [27,47]. This is possibly because the added adsorbent provided excessive active groups beyond the capacity to be accepted by the heavy metal ions, leading to a reduced number of ions adsorbed onto the unit weight of the adsorbent. While the hydrogel dosage is between 0.5 and 0.6 g, the change in adsorption capacity is relatively small. Therefore, the dosage of hydrogel in this work is 0.5 g. 

#### 2.2.6. Reusability

**Figure 9:**
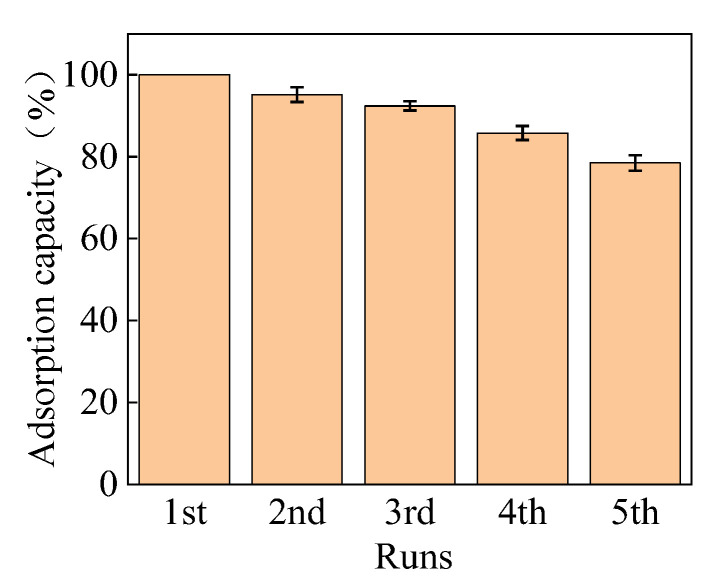
Reusability of the hydrogel for Cu^2+^ adsorption.

The PGA hydrogel absorbed with Cu^2+^ was regenerated by desorption of Cu^2+^ with ammonia water as the desorbent. Adsorption-desorption cycles were carried out to evaluate the reusability of the hydrogel for Cu^2+^ removal. The adsorption capacity of the first adsorption process was set as 100%, and the adsorption capacity ratio of each cycle versus the first cycle was calculated for the reusability evaluation [43]. As shown in Figure 9, the ratio decreased gradually, but it remained at 95% in the first run and remained at 78.5% in the fifth run, indicating that the hydrogels had excellent reusability. The reuse of the PGA hydrogel in metal ion removal reduces resource consumption and makes the material cost-effective, in contrast to the non-renewable PGA aqueous solution adsorbent [13].

#### 2.2.7. Adsorption of Other Heavy Metal Cations

**Figure 10:**
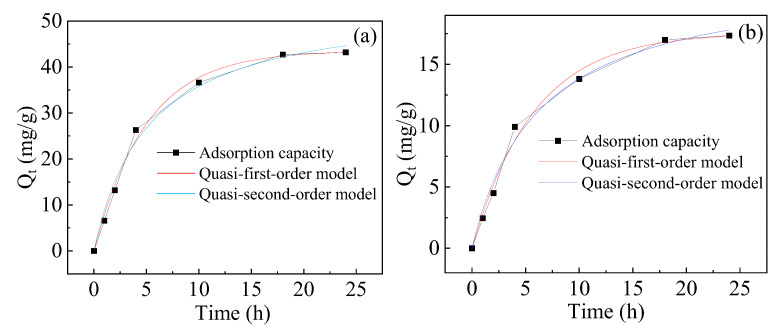
Adsorption kinetic of (**a**) Zn^2+^ and (**b**) Cr^2+^ on the hydrogel.

The adsorptions of Zn^2+^ and Cr^6+^ by the hydrogel were also investigated. The experiment was carried out by immersing 0.03 g hydrogel in Zn^2+^ (200 mg/L, pH 7) and Cr^6+^ (30 mg/L, pH 7) solution at 25 °C. As shown in Figure 10a,b, the adsorption capacities are 43.2 mg/g and 17.4 mg/g, respectively, illustrating the applicability of the hydrogel for the removal of Zn^2+^ and Cr^6+^.

The fitted curves of the adsorption kinetic equation and the fitted parameters are shown in Figure 10 and Table 4, respectively. Physical adsorption is also dominant in the adsorption process, because the adsorption process followed the pseudo-first-order kinetic better than the pseudo-second-order kinetic, based on the correlation coefficients (R^2^) of the equations. 

**Figure 11:**
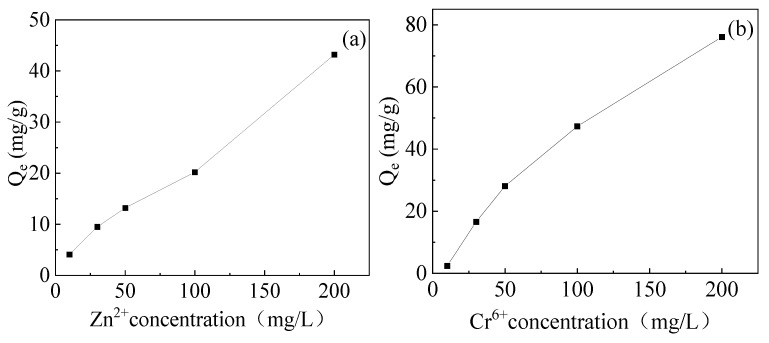

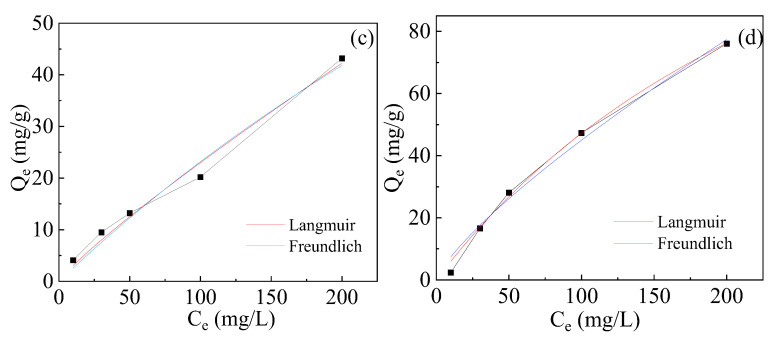
Adsorption isotherm of (**a**) Zn^2+^ and (**b**) Cr^2+^ and fitted curves of Langmuir and Freundlich adsorption isotherm models of (**c**) Zn^2+^ and (**d**) Cr^2+^.

The adsorption isotherm of Zn^2+^ and Cr^2+^ and fitted curves of the Langmuir and Freundlich adsorption isotherm models are shown in Figure 11. The parameters from two isotherm models are shown in Table 5, respectively. Based on the R^2^ values, the adsorption process is better suited to the Langmuir model than the Freundlich isotherm. Therefore, the adsorptions of Zn^2+^ and Cr^2+^ by hydrogel are also mainly through single-layer adsorption.

The thermodynamic parameters from the adsorption experiment of Zn^2+^ and Cr^2+^ at different temperatures are demonstrated in Table 6. The positive value of Δ*H*^0^ of adsorption indicates that the adsorption process is endothermic. The negative values of Δ*G*^0^ represent the spontaneous nature of adsorption.

### 2.3. Adsorption Mechanism

**Figure 12:**
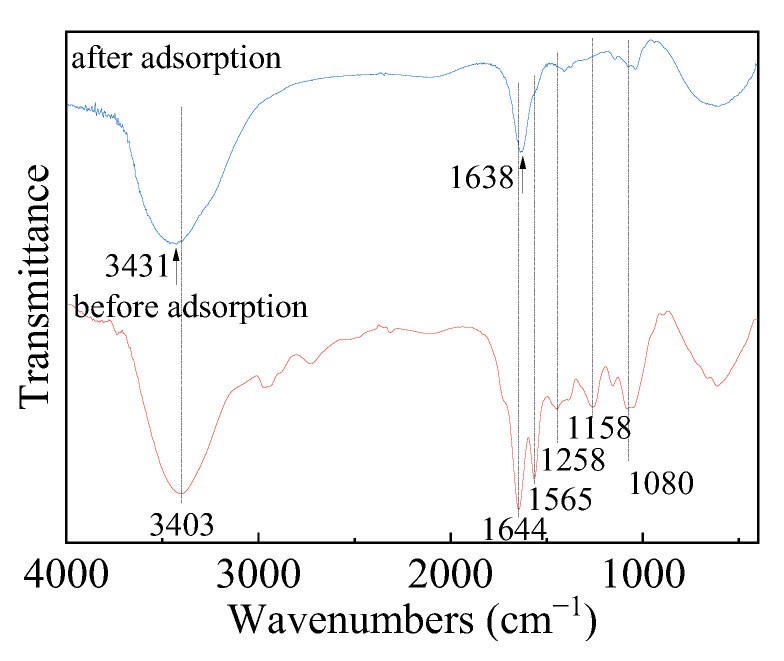
FTIR of the hydrogels before and after the adsorption.

To explore the adsorption mechanism, FTIRs of the hydrogels before and after the adsorption of Cu^2+^ were compared and are shown in Figure 12. Upon adsorption, the peaks at 3403 and 1644 cm^−1^ attributable to O–H and N–H vibrations shifted to 3431 and 1638 cm^−1^, respectively, indicating that the carboxylic hydroxyl (–COOH) and –NH functional groups participated in the adsorption of copper ions. In addition, the peak at 1565 cm^−1^ belonging to the N–H vibration, as well as the peaks at 1258, 1158, and 1080 cm^−1^ attributable to the stretching vibrations of C–O or C–N, weakened after the adsorption process, confirming that –COOH and –NH groups in the hydrogel were the main sites of Cu^2+^ adsorption. This is consistent with the results of the effect of solution pH on the adsorption capacity. The electron-donating –NH groups adsorb Cu^2+^ via the coordination interactions and –COO^−^ via electrostatic interactions.

**Figure 13:**
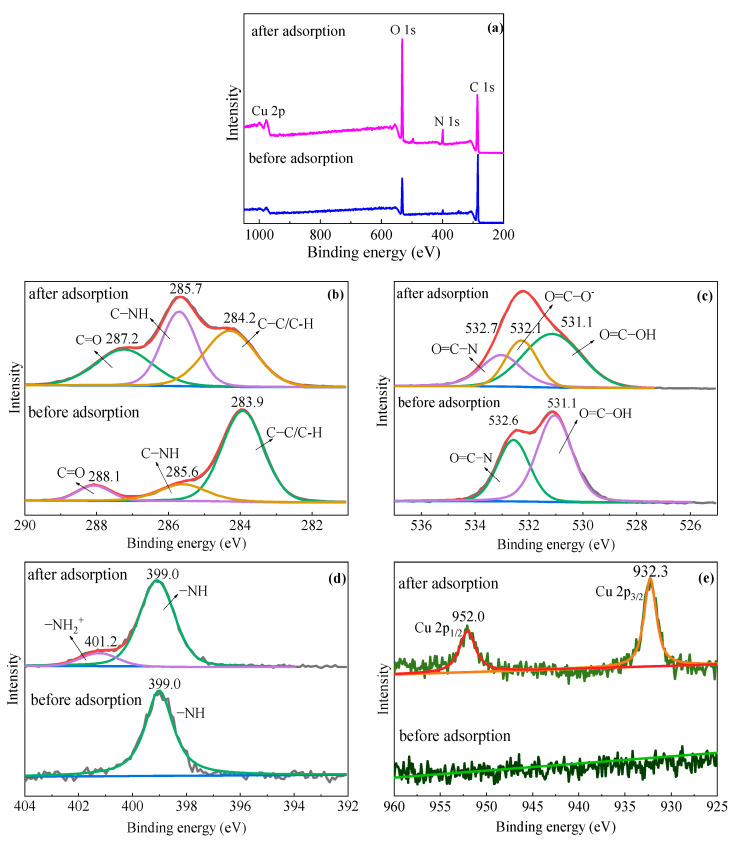
XPS spectra of PGA before and after metal adsorption: (**a**) survey spectra, (**b**) C1s spectra, (**c**) O1s spectra, (**d**) N1s spectra, and (**e**) Cu2p spectra.

To explore the adsorption mechanism further, XPS was also used to detect the chemical bonds on the hydrogel surface. Figure 13a shows the XPS survey scans and Figure 13b–e exhibit the high-resolution scans of C1s, O1s, N1s, and Cu2p spectra with their respective deconvolutions. In the C1s spectrum of hydrogel before adsorption, the peaks centered at 283.9, 285.6, and 288.1 eV are related to C–C/C–H bonds, C–NH, and C=O, respectively [48]. After adsorption, the peaks slightly shifted to 284.2, 285.7, and 287.2 eV, respectively, possibly due to the interaction with Cu^2+^ [29]. In the O1s spectrum of the hydrogel before adsorption, the peaks centered at 532.6 and 531.1 eV are related to O=C–N and O=C–OH, respectively [30,49]. After adsorption, the spectrum showed a new peak at 532.1 eV, attributed to the transformation of the carboxylic groups (–COOH) to carboxylates (–COO^−^) by the adsorption of Cu^2+^ [30]. This indicates that Cu^2+^ adsorbed on the hydrogel via the electrostatic interactions with –COO^−^ [1]. The N1s spectra of the hydrogel before adsorption exhibited peaks centered at 399.0 eV assigned to the N–H groups [29,49]. After adsorption, a peak centered at 401.2 eV belonging to –NH_2_^+^ appeared [29,49]. The H^+^ for protonation of –NH comes from the –COOH group on PGA and the deprotonated –COO^−^ anion binds to the Cu^2+^ ion, consistent with the results of the O1s spectra. As shown in Figure 13e, two peaks centered at 932.3 and 952.0 eV associated with Cu 2p_3/2_ and Cu 2p_1/2_ [50], respectively, appeared after adsorption, which was not observed before the adsorption. This indicates that Cu^2+^ was captured into the hydrogel.

## 3. Conclusions

A water-insoluble and recyclable PGA-based hydrogel was prepared for heavy metal removal. The hydrogel form of adsorbent made the adsorption operation simple. The adsorption capacity of the hydrogel for Cu^2+^ is 8.6 mg/g. It is similar to that of PVA and much lower than that of chitosan, modified chitosan, and cellulose. The adsorption is a single-layer physisorption and Cu^2+^ is adsorbed on the hydrogel via electrostatic interactions with –COO^−^ in addition to the coordination interactions with the –NH of PGA. Therefore, the adsorption capacity of the hydrogel for Cu^2+^ increased with the increase in solution pH with more –COO^−^ and the deprotonated –NH groups. The adsorption process is spontaneous and exothermic, and a low temperature is favorable to the process. The PGA-based hydrogel had good reusability and it made the hydrogel a cost-effective adsorption material for heavy metal ion removal from wastewater. However, the adsorption capacity of the PGA hydrogel shows a decrease in removal efficiency with cycles. In addition, there are other limitations; for example, the application of hydrogel-based adsorbent materials in heavy metal removal is limited to lab scale. Further research is required to scale up for a large-scale application. In addition, this research lacks the research of selectivity of the sorbent concerning copper in the presence of zinc, chromium, and other heavy metals. The present research is also confined to the removal of three kinds of heavy metal ions. More research should be undertaken targeting multiple heavy metals.

## 4. Materials and Methods

### 4.1. Materials and Instruments

PGA (molecular weight 700,000) was purchased from Shanghai Yika Biotechnology Co., Ltd. (Shanghai, China). PAMAM was obtained from Weihai Chenyuan Molecular New Materials Co., Ltd. (Weihai, China). N-(3-dimethylaminopropyl)-N-ethylcarbodiimide hydrochloride (EDC) was obtained from Hebei Bailingwei Ultrafine Materials Co., Ltd. (Langfang, China). Sodium diethyldithiocarbamate trihydrate (SDDT) came from Shanghai Aladdin Biochemical Technology Corporation (Shanghai, China). N-hydroxysuccinimide (NHS), potassium bromide (KBr), ammonia water, and copper chloride (CuCl_2_) were all purchased from Sinopharm Chemical Reagents Co., Ltd. (Shanghai, China). Deionized water was used in the experiment.

### 4.2. Characterizations

The absorbances of Cu^2+^ solutions were measured using an ultraviolet-visible (UV-Vis) spectrophotometer (TU-1950, Beijing Puxi General Instrument Co., Ltd., Beijing, China). The concentrations of Zn^2+^ and Cr^6+^ solutions were determined by an inductively coupled plasma spectrometer (ICP2060T, Jiangsu Skyray Instrument Co., Ltd., Kunshan, China). Fourier transform infrared spectrum (FTIR) was recorded by an infrared spectrometer (FTIR-650, Tianjin Gangdong Science and Technology Development Co., Ltd., Tianjin, China). The surface morphologies of hydrogels were examined by scanning electron microscope (SEM) (ZEISS Gemini 300, China Academy of Sciences Brain Science and Intelligent Technology Innovation Center, Shanghai, China). X-ray photoelectron spectroscopy (XPS) was performed using a Thermo Scientific K-Alpha spectrometer (Thermo Fisher Scientific Inc., Waltham, MA, USA). The surface area was determined with the Brunauer-Emmett-Teller (BET) method using a fully automatic specific surface area analyzer (APSP 2460, Micromeritics Instrument Corp., Norcross, GA, USA).

### 4.3. Preparation of Hydrogel

Firstly, PGA aqueous solution was obtained by dissolving 0.15 g PGA in 3 mL water by stirring for 30 min using an ultrasonic instrument. Secondly, the PAMAM aqueous solution was prepared by adding 100 μL of 0.21 g/mL PAMAM methanol solution into 1 mL water and shaking. Thirdly, PGA and PAMAM solutions were mixed evenly by stirring for 30 min. Finally, 1 mL aqueous solution containing 0.08 g EDC and 0.08 g NHS was added to the mixture and the stirring continued until the hydrogel formed. The hydrogel was freeze-dried for use.

### 4.4. Determination of Cu^2+^ Concentration

SDDT is used as a chromophoric reagent for UV-Vis spectrophotometric determination of Cu^2+^ concentration [43]. Briefly, 1 mL SDDT solution (0.2%, *w*/*w*) was added to 5 mL Cu^2+^ solutions of different concentrations (0, 0.5, 1.0, 2.0, 4.0, 6.0, 8.0, 10.0 mg/L). The solution absorbance at 447 nm was recorded to create a standard curve of absorbance against concentration. The samples of unknown concentrations were tested for their absorbance and the concentrations were determined using the standard curve.

### 4.5. Adsorption Kinetics

The static adsorption of Cu^2+^ by the hydrogel was carried out by immersing 0.5 g ± 5 mg hydrogel (a cylindrical body with a diameter of 10 mm and a height of 15 mm) into 100 mL Cu^2+^ solution (100 mg/L, pH 7) at 25 °C. At a given time interval, the solution absorbance at 447 nm was measured to determine the residual Cu^2+^ concentration. The adsorption capacity of the hydrogel at time t (*Q_t_*, mg/g) and equilibrium (*Q_e_*, mg/g) were calculated using Equations (5) and (6), respectively.

(5) $$Q_{t}=\frac{(C_{0}-C_{t})V}{m}$$

(6) $$Q_{e}=\frac{(C_{0}-C_{e})V}{m}$$

where *C*_0_ (mg/L) and *C_e_* (mg/L) represent the initial and equilibrium concentration of Cu^2+^, respectively; *C_t_* (mg/L) is the residual Cu^2+^ concentration at time t; *V* (L) represents the solution volume; *m* (g) is the adsorbent weight.

### 4.6. Adsorption Isotherms

Quantities of 0.5 g ± 5 mg hydrogel were immersed in 100 mL Cu^2+^ solution of different concentrations (10~100 mg/L, pH 7) at 25 °C for the static adsorption of Cu^2+^ and allowed to reach a state of adsorption equilibrium.

### 4.7. Adsorption Thermodynamics

Quantities of 0.5 g ± 5 mg hydrogel were put into 100 mL Cu^2+^ solution (100 mg/L, pH 7), at 25 °C, 35 °C, and 45 °C, for the static adsorption of Cu^2+^ to an adsorption equilibrium. The experimental data were analyzed to calculate the thermodynamic parameters according to thermodynamic Equations (7)–(10), respectively.

(7) ΔG=−RTlnK

(8) ΔG=ΔH−TΔS

(9) $$\ln K=-\frac{\Delta H}{R}\cdot \frac{1}{T}+\frac{\Delta S}{R}$$

(10) $$K=\frac{Q_{e}}{C_{e}}$$

where Δ*G* (J/mol), Δ*H* (J/mol), and Δ*S* represent the changes in Gibbs free energy, enthalpy, and entropy, respectively. *K* (L/mol) is the equilibrium constant and *R* represents the universal gas constant. *T* (K) is the absolute temperature. 

### 4.8. Effect of the Solution pH on Adsorption

To investigate the effect of solution pH on Cu^2+^ adsorption, 0.5 g ± 5 mg hydrogel was immersed in 100 mL Cu^2+^ solutions at different pH (10 mg/L, pH 3~7) at 25 °C for the static adsorption of Cu^2+^ to a state of adsorption equilibrium.

### 4.9. Effect of the Mass of the Adsorbent on Adsorption

To investigate the effect of the mass of the adsorbent on Cu^2+^ adsorption, various gram quantities of hydrogel were immersed in 100 mL Cu^2+^ solutions (10 mg/L, pH 7) at 25 °C for the static adsorption of Cu^2+^ to a state of adsorption equilibrium.

### 4.10. Reusability

To investigate the recyclability performance of the adsorbent, the hydrogel adsorbed with Cu^2+^ was recovered from the solution and desorbed with 0.1 mol/L ammonia solution for 4 h for regeneration. The hydrogel was then treated with the deionized water until neutrality for the next adsorption process. 

### 4.11. Swelling Behavior of the Hydrogels

A quantity of 0.36 g hydrogel was immersed in 100 mL water until reaching a swelling equilibrium; the swelling ratio (*W*) of the hydrogel was calculated according to Equation (11).

(11) $$W=\frac{m_{s}}{m_{d}}$$

where *m_s_* (g) and *m_d_* (g) are the weights of the swollen and freeze-dried hydrogel, respectively. 

## Figures and Tables

**Table 1:** Kinetic parameters of pseudo-first-order and pseudo-second-order models for Cu^2+^ adsorption onto the hydrogel.

*Q_e_* (mg/g)	Pseudo-First-Order			Pseudo-Second-Order		
	*q_e_*_1_ (mg g^−1^)	*k*_1_ (min^−1^)	R^2^	*q_e_*_2_ (mg g^−1^)	*k*_2_ (g mg^−1^ min^−1^)	R^2^
8.6 ± 0.1	8.64	9.04 × 10^−2^	0.9989	8.84	0.2974	0.9261

**Table 2:** Isotherm parameters of Cu^2+^ adsorption onto hydrogel.

Langmuir Model			Freundlich Model		
*Q_m_* (mg g^−1^)	*K_L_* (L mg^−1^)	R^2^	*K_F_* (L^n^ mg^n−1/n^ g^−1^)	1/n	R^2^
21.41	0.0069	0.9863	0.2833	0.7466	0.9781

**Table 3:** Thermodynamic parameters for Cu^2+^ adsorption onto hydrogel.

Δ*H*^0^ (kJ·mol^−1^)	Δ*S*^0^ (kJ·mol^−1^·K^−1^)	Δ*G*^0^ (kJ·mol^−1^)			R^2^
		298 K	318 K	328 K	
−1792.42	−5.4252	−175.70	−67.20	−12.95	0.9999

**Table 4:** Kinetic parameters of pseudo-first-order and pseudo-second-order models for the adsorption of Zn^2+^ and Cr^6+^ onto the hydrogels.

Metal Ions	*Q_e_* (mg/g)	Pseudo-First-Order			Pseudo-Second-Order		
		*q_e_*_1_ (mg g^−1^)	*k*_1_ (min^−1^)	R^2^	*q_e_*_2_ (mg g^−1^)	*k*_2_ (g mg^−1^ min^−1^)	R^2^
Zn^2+^	43.2	43.52	0.2011	0.9938	54.1	0.0035	0.9881
Cr^6+^	17.35	17.57	0.1735	0.9910	22.41	0.0071	0.9883

**Table 5:** Isotherm parameters of Zn^2+^ and Cr^2+^ adsorption onto hydrogels.

Heavy Metal Ions	Langmuir Model			Freundlich Model		
	*Q_m_* (mg g^−1^)	*K_L_* (L mg^−1^)	R^2^	*K_F_* (L^n^ mg^n−1/n^ g^−1^)	1/n	R^2^
Zn^2+^	212.3	0.0012	0.9994	0.4039	0.8774	0.9819
Cr^6+^	198.63	0.0031	0.9939	1.2225	0.7829	0.9850

**Table 6:** Thermodynamic parameters for metal ion adsorption onto hydrogel.

Heavy Metal Ions	Δ*H*^0^ (kJ·mol^−1^)	Δ*S*^0^ (kJ·mol^−1^·K^−1^)	Δ*G*^0^ (kJ·mol^−1^)			R^2^
			298 K	308 K	318 K	
Zn^2+^	0.12238	0.00046	−0.0147	−0.0193	−0.024	0.9863
Cr^6+^	0.3635	0.0013	−0.0477	−0.0615	−0.0729	0.9999

## Data Availability

Data are contained within the article.
